# The food retail environment and area deprivation in Glasgow City, UK

**DOI:** 10.1186/1479-5868-6-52

**Published:** 2009-08-06

**Authors:** Laura Macdonald, Anne Ellaway, Sally Macintyre

**Affiliations:** 1 MRC Social & Public Health Sciences Unit, 4 Lilybank Gardens, Glasgow, G12 8RZ, UK

## Abstract

It has previously been suggested that deprived neighbourhoods within modern cities have poor access to general amenities, for example, fewer food retail outlets. Here we examine the distribution of food retailers by deprivation in the City of Glasgow, UK.

We obtained a list of 934 food retailers in Glasgow, UK, in 2007, and mapped these at address level. We categorised small areas (data zones) into quintiles of area deprivation using the 2006 Scottish Index of Multiple Deprivation Income sub-domain score. We computed mean number of retailers per 1000 residents per data zone, and mean network distance to nearest outlet from data zone centroid, for all retailers combined and for each of seven categories of retailer separately (i.e. bakers, butchers, fruit and vegetable sellers, fishmongers, convenience stores, supermarkets and delicatessens).

The most deprived quintile (of areas) had the greatest mean number of total food retailers per 1000 residents while quintile 1 (least deprived) had the least, and this difference was statistically significant (Chi-square p < 0.01). The closest mean distance to the nearest food retailer was within quintile 3 while the furthest distance was within quintile 1, and this was also statistically significant (Chi-square p < 0.01). There was variation in the distribution of the seven different types of food retailers, and access to amenities depended upon the type of food retailer studied and whether proximity or density was measured. Overall the findings suggested that deprived neighbourhoods within the City of Glasgow did not necessarily have fewer food retail outlets.

## Background

The prevalence of obesity is increasing in industrialised countries. Almost a quarter of adults in the UK are now classified as obese [1] with higher rates among low income groups (particularly women) [2]. The principal cause of obesity is an imbalance between energy intake and energy expenditure. Although a range of factors may contribute to rising obesity levels [3], it has been suggested that more focus should be directed towards an ecological approach to the obesity epidemic and that: 'Understanding, measuring, and altering the "obesogenic" environment is critical to success' [4]. Obesogenic environments are those which promote excessive food intake and discourage physical activity. A growing number of studies explore the potential contribution of the local food retail environment [5-11]. The findings of these studies varied depending on the type of resource(s) measured and the Country, State or City in which the study was based. Studies, based in regions of the US, found a smaller number of supermarkets [6,12,13] and grocery stores [7], within lower income and predominantly black areas, while other US studies found that, although more disadvantaged areas had fewer large supermarkets, they had a greater number of smaller convenience and grocery stores [10,14-16]. Some studies in Ontario, Canada, and in Melbourne, Australia, found that more advantaged areas had more supermarkets [8,17,9,18], while other Canadian studies, and also studies in New Zealand, found that more disadvantaged areas had more supermarkets [19,11-22]. Within the UK, there is limited literature on the geographical distribution of food outlets [23]. The results of a study in Newcastle-upon-Tyne showed that in general food shops were located in less socio-economically deprived areas, while specifically general stores, multiples and delicatessens tended to be in less deprived areas, and local discount stores, ethnic food stores, freezer centres, fishmongers, and greengrocers tended to be in more deprived areas [24]. Previous research in the Greater Glasgow Health Board area found that more deprived neighbourhoods had better access to food retail outlets [25]. Given the diversity in findings between various studies, and the need to update previous work with exploration of Glasgow's current food retail environment, we aim to examine the location of food retailers, by small area deprivation, and establish whether a pattern by deprivation for food retail outlets exists. We also see the importance in investigating patterns by deprivation for a range of different types of food retail outlet (e.g. butcher, baker, fishmonger etc) as existing research tends to focus on one or two specific type(s) of food retail outlet, such as supermarkets or grocery stores.

## Methods

A list of food retailers in Glasgow City with postcodes was obtained from Glasgow City Council, as of 2007. The list is held by the Council for licensing, inspection and planning purposes. The list included 7 categories of food retailer: bakers, butchers, fruit and vegetable (F&V), fishmongers, convenience stores, supermarkets and delicatessens (delis). A convenience store sells staple items such as milk and bread, in addition to, a limited selection of fruit and vegetables, tinned produce, snack food (e.g. crisps, chocolate and sweets), soft drinks, newspapers, cigarettes and may hold a liquor license.

Look-up tables were used to link the unit postcodes of each retail outlet to Scottish data zones. The data zone is the key small-area statistical geography in Scotland [26]. The data zone geography covers the whole of Scotland and nests within local government boundaries. Data zones are groups of 2001 Census output areas and the majority have populations of between 500 and 1,000 residents. Where possible, they have been made to respect physical boundaries and natural communities. They have a regular shape and, as far as possible, contain households with similar social characteristics.

There are 694 data zones in the Glasgow City Council boundary, with a mean population of 832 (range 248 – 2243) and a mean area of 25.2 hectares [26]. In 2006 Glasgow City had a population of around 580,690 people, and covered approximately 17,730 hectares [26]. For each data zone we obtained the 2006 Scottish Index of Multiple Deprivation (SIMD) Current Income sub-domain score [27] (the 2006 SIMD was used as a SIMD for 2007 or 2008 has not been developed). The SIMD is a publicly available continuous measure of compound social and material deprivation, calculated using data such as employment, welfare benefits, health, education and housing for each data zone. We chose not to use the full index since it includes health variables and access to services, so there might have been some circularity in investigating whether it predicted access to resources. The Current Income sub-domain is based on numbers of residents claiming a range of financial welfare benefits (e.g. Income Support, Guaranteed Pension Credit, Job Seekers Allowance [27]). We divided SIMD scores for Glasgow into quintiles (Q1 = least deprived, Q5 = most deprived). Quintiles 1–4 contain 139 data zones each while quintile 5 includes 138 data zones). We calculated quintiles separately for the Glasgow city area (as opposed to using the existing Scotland wide categories) because deprived neighbourhoods using the national classification are overrepresented in Glasgow. The average area of data zones differed slightly across these quintiles, being greatest (28.6 Ha) in Q4 and least (22.3 Ha) in Q5.

For all food retailers together and each separately, we calculated the mean number of retailers per thousand population; and the mean network distance in metres from the centroid of each data zone to the nearest retailer.

We used population data from the General Register for Scotland's 2006 small area estimates for each data zone [26]. to calculate the density of each retailer per 1000 people per quintile. (Areas without any outlets were also included). Comparison of density between quintiles was determined by ANOVA using SPSS version 14.0.

Network analysis (i.e. finding the shortest path between two locations on a road network) was carried out for each retailer using ArcMap v9.1. Street maps (including point addresses) were obtained from UK Ordnance Survey [28]. Every retailer was geocoded by unit postcode. Network analysis was undertaken to find the network distance in metres from the centroid of each data zone to the nearest retailer type and we then calculated the mean distance to the nearest retailer within each SIMD quintile. Comparison between quintiles of mean distances to retailers was determined by ANOVA in SPSS v14.0.

In addition, analyses were carried out combining bakers, butchers, F&V sellers, fishmongers and convenience stores with supermarkets, as bread, meat, fruit and vegetables, fish and snack products can also be purchased at supermarkets.

## Results

**Figure 1:**
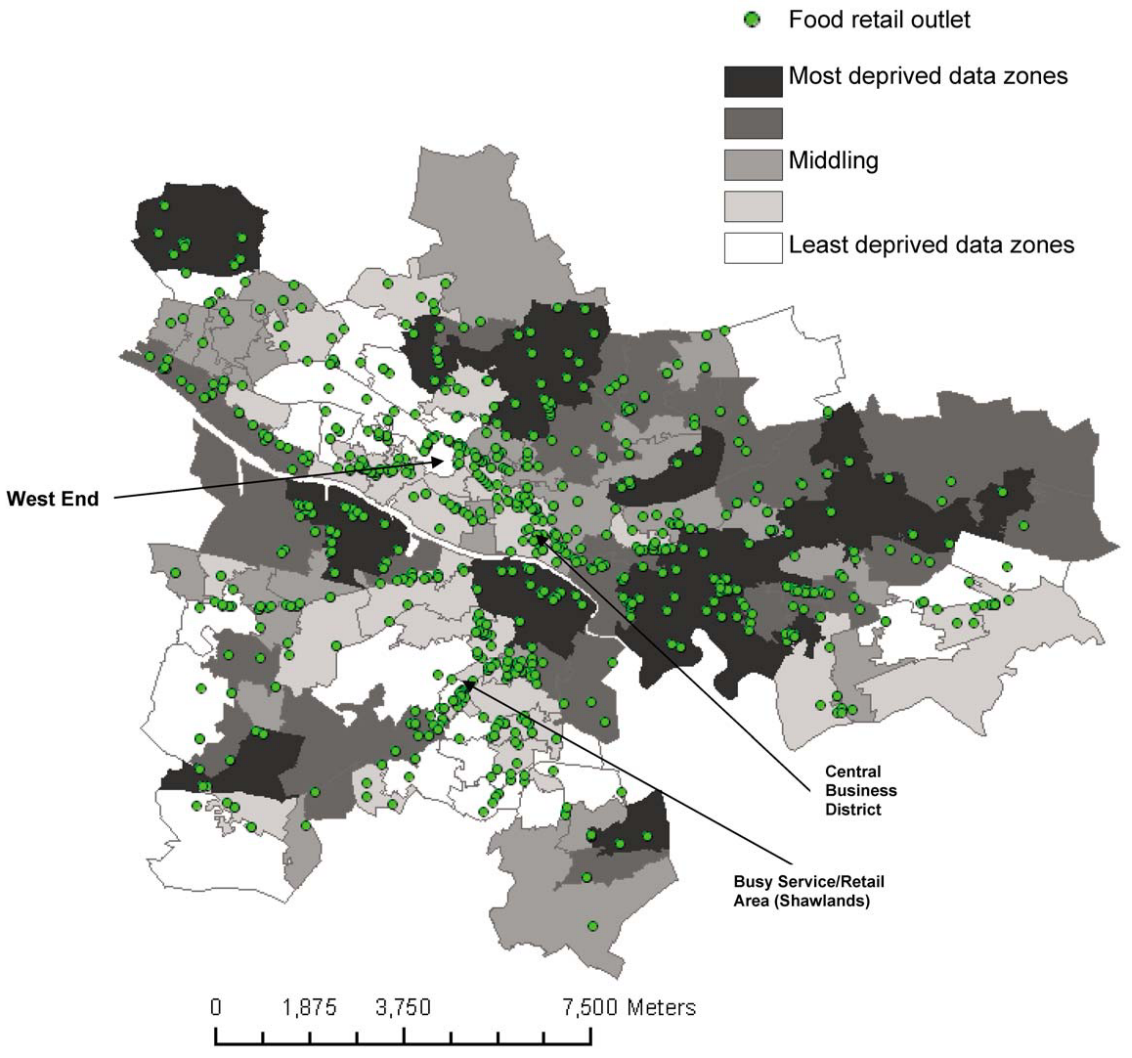
**Glasgow City: Food retail outlets by Income deprivation by data zone**.

The analysis included 934 food retailers (see figure 1); 61 bakers, 94 butchers, 36 F&V sellers, 16 fishmongers, 637 convenience stores, 68 supermarkets and 22 delis.

There were no statistically significant differences between mean number of bakers, F&V sellers, fishmongers, supermarkets or delis per 1000 residents (see table 1). The least deprived quintile (Q) showed the smallest mean number of total food retailers per 1000 residents (0.99), while there was little difference between Q2, Q3 and Q5 (≈1.9 each) (p < 0.01). Q1 and Q4 showed the smallest mean number of butchers (0.07, 0.09 respectively), while there was little variation between the other quintiles (≈0.2 each) (p < 0.05). The least deprived quintile showed the smallest mean number of convenience stores (0.64), while Q2 and Q5 showed the highest numbers (≈1.3 each) (p < 0.01) (see table 1).

**Table 1:** Per SIMD Income quintile: number of retailers; mean number per 1,000 residents; mean distance to nearest

		**Number**	**Mean N per 1000 residents**	**Mean distance (metres) to nearest retailer**
***All food retailers***				
**SIMD Quintile**	**1 Least deprived**	118	0.99	504
	**2**	227	1.87	379
	**3 Middling**	218	1.89	361
	**4**	163	1.44	412
	**5 Most Deprived**	208	1.94	445
				
	**Total**	934	1.63	420
		Chi^2^	*F = 3.46 p = 0.008*	*F = 4.47 p = 0.001*
		Linearity	*F = 4.45 p = 0.035*	F = 0.99 p = 0.319

***Bakers***				
**SIMD Quintile**	**1 Least deprived**	12	0.09	1329
	**2**	12	0.09	1238
	**3 Middling**	15	0.13	1383
	**4**	8	0.07	1536
	**5 Most Deprived**	14	0.11	1559
				
	**Total**	61	0.10	1409
		Chi^2^	F = 0.62 p = 0.651	F = 2.27 p = 0.060
		Linearity	F = 0.04 p = 0.852	*F = 6.97 p = 0.008*

***Butchers***				
**SIMD Quintile**	**1 Least deprived**	9	0.07	1204
	**2**	29	0.24	1090
	**3 Middling**	22	0.20	1047
	**4**	11	0.09	1102
	**5 Most Deprived**	23	0.22	1113
				
	**Total**	94	0.16	1111
		Chi^2^	*F = 2.99 p = 0.018*	F = 0.91 p = 0.456
		Linearity	F = 1.09 p = 0.297	F = 0.79 p = 0.375

***F&V sellers***				
**SIMD Quintile**	**1 Least deprived**	5	0.04	1746
	**2**	8	0.06	1716
	**3 Middling**	7	0.06	1611
	**4**	4	0.04	1512
	**5 Most Deprived**	12	0.12	1558
				
	**Total**	36	0.06	1629
		Chi^2^	F = 1.51 p = 0.198	F = 1.44 p = 0.220
		Linearity	F = 2.12 p = 0.146	*F = 4.80 p = 0.029*

***Fishmongers***				
**SIMD Quintile**	**1 Least deprived**	1	0.01	2401
	**2**	3	0.02	2455
	**3 Middling**	5	0.05	2547
	**4**	3	0.03	2732
	**5 Most Deprived**	4	0.03	3076
				
	**Total**	16	0.03	2641
		Chi^2^	F = 0.78 p = 0.541	*F = 3.56 p = 0.007*
		Linearity	F = 0.88 p = 0.350	*F = 12.61 p = 0.000*

***Convenience stores***				
**SIMD Quintile**	**1 Most Affluent**	75	0.64	538
	**2**	153	1.28	408
	**3 Middling**	145	1.25	381
	**4**	126	1.13	434
	**5 Most Deprived**	138	1.31	486
				
	**Total**	637	1.12	449
		Chi^2^	*F = 3.65 p = 0.006*	*F = 4.57 p = 0.001*
		Linearity	*F = 6.75 p = 0.010*	F = 0.69 p = 0.403

***Supermarkets***				
**SIMD Quintile**	**1 Most Affluent**	10	0.08	1098
	**2**	14	0.11	1078
	**3 Middling**	20	0.17	1215
	**4**	10	0.08	1350
	**5 Most Deprived**	14	0.12	1569
				
	**Total**	68	0.11	1262
		Chi^2^	F = 0.74 p = 0.568	*F = 8.02 p = 0.000*
		Linearity	F = 0.16 p = 0.692	*F = 28.68 p = 0.000*

***Delicatessens***				
**SIMD Quintile**	**1 Most Affluent**	5	0.05	1812
	**2**	6	0.05	1679
	**3 Middling**	5	0.04	1948
	**4**	1	0.01	2174
	**5 Most Deprived**	5	0.04	2136
				
	**Total**	22	0.04	1950
		Chi^2^	F = 0.88 p = 0.474	*F = 4.42 p = 0.002*
		Linearity	F = 0.61 p = 0.433	*F = 13.04 p = 0.000*

There were no significant differences between quintiles in terms of mean distance in metres (m) to the nearest baker, butcher, or F&V seller (see table 1). The distance to the nearest food retailer (any) was smallest within Q3 (361 m) and Q2 (379 m) while the distance was greatest within the least deprived quintile (504 m) (p < 0.01). Convenience stores were nearer within Q3 (381 m) and Q2 (408 m) and further within Q1 (538 m) (p < 0.01). With increasing deprivation, the distance to the nearest fishmonger increased (p < 0.01). There was a general increase in distance to the nearest deli, and nearest supermarket, with increasing deprivation (p < 0.01 and p < 0.001 respectively) (see table 1).

When we combined specific categories of food retail outlet with supermarkets, the findings did not differ greatly from those above (results not shown, available from authors).

## Conclusion

We found that in terms of density of all food retail outlets/distance to the nearest food retail outlet, the least deprived quintile was the least well served, while the most deprived quintile had the greatest density, and quintile 3 the closest mean distance. Findings varied when each category of food outlet was analysed separately. Overall the findings suggest that deprived neighbourhoods within the City of Glasgow do not necessarily have poorer access, and more advantaged neighbourhoods better access, to food retail outlets. In keeping with other studies, findings varied depending on the type of food outlet measured. It has been shown in previous research within Glasgow that various different types of resources and amenities were more likely to be located within the second least deprived quintile, such as restaurants, cafes, dental practices, banks, ATMs etc [29,30]. We found that butchers and convenience stores had a greater density within quintile 2, and the nearest supermarket and deli was closest within quintile 2. This quintile tends to be closer to the central business district, other retail, office and service hubs (e.g. the West End, Shawlands) (see figure 1), which would be busy both during the day and evening, and therefore have large numbers of potential customers.

There are several caveats relating to our the findings. We did not check the accuracy of the database of food outlets obtained from the Council as outlets were too numerous to do so. However a study within Glasgow surveyed samples of food stores in 1997 (N = 325) and 2007 (N = 508) (data obtained from Glasgow City Council) and found by visiting stores in person that 87% and 88%, respectively, were confirmed as open and trading as food stores (Cummins S, Macintyre S: How accurate are secondary data sources on the neighbourhood food environment?, submitted). This proportion did not vary significantly by the level of deprivation (DEPCAT Score) within the postcode in which food stores were located at either time point. Although we looked at access to food retail outlets in terms of density and proximity we did not explore quality, price, or nutritional value of food products within each outlet. These factors require more detailed study, which is being carried forward in current research (i.e. co-workers are comparing the price, availability and quality of a controlled number of foodstuffs by area deprivation from a sub-sample of food outlets within Glasgow, but are not studying the distribution of all outlets by deprivation), but is beyond the scope of this present study. We can not assume that every food store within each of the categories (e.g. convenience stores, supermarkets etc) are all equal, but they do share similar characteristics which make it viable to group them, e.g. supermarkets are large chain retail stores (between 500–2500 square metres approx.) operating on a self-service basis, selling groceries, produce, meat, bakery and dairy, and some non-food goods [31] while convenience stores are smaller and have a limited range of generally higher-priced produce [32]. It must also be noted that proximity does not always predict use, and may not be as important in areas where car access is high. Car access within the UK is greater among higher social classes so having a food outlet nearby may not be as important to these residents [33]. People may use food retail outlets near their place of work, study or child's school. Home delivery from supermarkets may also be an option for weekly shopping, and also takeaway food, or use of mobile vans selling, for example, fish produce. We can not assume that people's food shopping takes place within the boundaries of an administratively defined area. However, we did use relatively small areas which are designed to respect physical boundaries and natural communities, and comparing findings using different administrative areas could be undertaken in future more in depth study. In spite of these issues we feel that our study has contributed to the literature exploring neighbourhood food retail environment and potential variations by deprivation, and provided a relatively up-to-date picture of food retailing within the City of Glasgow.

## Competing interests

The authors declare that they have no competing interests.

## Authors' contributions

LM did the mapping and data analysis, and wrote successive drafts of the paper. AE and SM contributed to the conception of the paper and discussion of the analysis and commented on drafts. All authors read and approved the final draft. LM is guarantor.
